# Integrative transcriptome analysis identifies a crotonylation gene signature for predicting prognosis and drug sensitivity in hepatocellular carcinoma

**DOI:** 10.1111/jcmm.70083

**Published:** 2024-10-20

**Authors:** Bailu Yang, Fukai Wen, Yifeng Cui

**Affiliations:** ^1^ Department of Hepatic Surgery The First Affiliated Hospital of Harbin Medical University Harbin China; ^2^ Key Laboratory of Hepatosplenic Surgery, Ministry of Education The First Affiliated Hospital of Harbin Medical University Harbin China

**Keywords:** hepatocellular carcinoma, lysine crotonylation‐related genes, prognostic characteristics, risk module

## Abstract

Hepatocellular carcinoma (HCC) stands as the most prevalent and treatment‐resistant malignant tumour, characterized by a dismal prognosis. Croton acylation (CA) has recently gained attention as a critical factor in cancer pathogenesis. This study sought to rapidly identify prognostic features of HCC linked to CA. Differential analysis was conducted between tumour tissues and adjacent non‐tumour tissues in the TCGA‐LIHC and GSE76427 datasets to uncover differentially expressed genes (DEG1 and DEG2). The intersection of DEG1 and DEG2 highlighted DEGs with consistent expression patterns. Single‐sample gene set enrichment analysis scores were calculated for 18 lysine crotonylation‐related genes (LCRGs) identified in prior research, showing significant differences between tumour and normal groups. Subsequently, weighted gene co‐expression network analysis was employed to identify key module genes correlated with the LCRG score. Candidate genes were identified by overlapping consistently expressed DEGs with key module genes. Prognostic features were identified, and risk scores were determined via regression analysis. Patients were categorized into risk groups based on the optimal cutoff value. Gene set enrichment analysis (GSEA) and immunoassays were also performed. The prognostic features were further validated using reverse transcription‐quantitative polymerase chain reaction (RT‐qPCR). A total of 88 candidate genes were identified from 1179 consistently expressed DEGs and 4200 key module genes. Seven prognostic features were subsequently identified: TMCO3, RAP2A, ITGAV, ZFYVE26, CHST9, HMGN4, and KLHL21. GSEA revealed that DEGs between risk groups were primarily associated with chylomicron metabolism, among other pathways. Additionally, activated CD4+ T cells demonstrated the strongest positive correlation with risk scores, and most immune checkpoints showed significant differences between risk groups, with ASXL1 exhibiting the strongest correlation with risk scores. The Tumour Immune Dysfunction and Exclusion score was notably higher in the high‐risk group. Moreover, in both the TCGA‐LIHC and ICGC‐LIRI‐JP datasets, the expression of other prognostic features was elevated in tumour tissues, with the exception of CHST9. RT‐qPCR confirmed the increased expression of TMCO3, RAP2A, ITGAV, ZFYVE26, and HMGN4. This study establishes a risk model for HCC based on seven crotonylation‐associated prognostic features, offering a theoretical framework for the diagnosis and treatment of HCC.

## 1 INTRODUCTION

Hepatocellular carcinoma (HCC) ranks as the fourth leading cause of cancer‐related mortality worldwide and is the sixth most prevalent malignancy, presenting a substantial threat to global health.^1^ In impoverished regions and areas with limited awareness of liver diseases, partial hepatectomy is often neglected due to insufficient screening and delayed detection at various middle and late stages, resulting in elevated mortality rates.^2^ HCC constitutes 90% of primary liver cancers, with its progression influenced by numerous factors, including the immune system.^3^ According to GLOBOCAN 2020, new cases and deaths from liver cancer are projected to rise by 58.6% and 60.9%, respectively, by 2040.^4^ The disease is linked to several risk factors, such as hepatitis C virus, aflatoxin‐contaminated diets, hepatitis B virus, smoking, excessive alcohol consumption, obesity, parasitic infections, and type 2 diabetes.^5^ Treatment options for HCC include liver transplantation, hepatectomy, ablation therapy, radiotherapy, TACE, and systemic antitumor therapy. Although advancements have led to significant reductions in technical and general complications, such as infections, tumour recurrence remains a pressing concern.^6^ Clinically, patients with tumours ≤2 cm, AFP‐negative tumours, or those without obvious symptoms are frequently overlooked. Moreover, there is no consensus on the specific pathogenesis of liver cancer.^7^ The development of liver cancer is a complex, multifactorial, and multistep process, involving genetics, environmental influences, lifestyle factors, and other variables. For instance, hepatitis B virus infection, chronic alcohol consumption and aflatoxin exposure are all critical risk factors. However, the exact mechanisms by which these factors interact to trigger HCC remain elusive.^8^ This ambiguity in pathogenesis complicates the diagnosis of liver cancer. Thus, the exploration of novel oncological markers for HCC could significantly enhance diagnostic accuracy and provide new therapeutic targets.

Crotonylation, a recently identified acylation modifier, has garnered significant attention over the past decade, with thousands of crotonylation sites identified in both histones and non‐histone proteins. Closely related to acetylation, crotonylation shares many of the same enzymes and plays a pivotal role in regulating various biological processes, including gene expression, cellular metabolism, DNA damage repair, stem cell differentiation and reproductive regulation. Its involvement extends to critical cellular functions such as gene expression, spermatogenesis and the cell cycle.^9^ Emerging evidence suggests that crotonylation is integral to the pathogenesis of a wide array of diseases, ranging from depression to cancer.^10^ For instance, crotonylation influences spermatogenesis by modulating the chromodomain Y‐like protein (CDYL). Moreover, studies in 2021 linked crotonylation to cardiac homeostasis and embryonic development.^11^ Crotonylation has also been associated with several physiological processes, including protein localization, RNA processing, regulation of protein activity, chromatin reorganization, and nucleotide metabolism.^12^ The primary focus of current research on crotonylation lies in its regulation of gene expression and protein function. Crotonyl‐CoA, the donor of the crotonyl group, is generated through fatty acid metabolism and can be transferred to lysine residues on proteins by crotonyltransferases, thereby altering protein function and interactions, which may impact cellular signalling and metabolism.^13^ In HCC, crotonylation appears to influence key cellular processes such as proliferation, apoptosis and angiogenesis. Specifically, non‐histone crotonylation has been implicated in the regulation of HCC metastasis and invasion, with evidence showing that crotonylation enhances cell invasion through the SEPT2‐K74‐P85α‐AKT pathway. Elevated SEPT2‐K74 crotonylation correlates with poor prognosis and high recurrence rates in patients with HCC, underscoring crotonylation's novel role in promoting HCC metastasis.^14^ Additionally, researchers have identified specific crotonyltransferases and their targets in HCC cells, offering insights into crotonylation's potential role in HCC pathogenesis.^15^ Although the understanding of crotonylation's role in HCC remains incomplete, existing evidence indicates its significant involvement in the disease's pathogenesis. Further research is essential to fully elucidate the mechanisms by which crotonylation contributes to HCC and to assess its potential as a therapeutic target.

This study utilized public databases related to HCC and crotonylation‐associated genes to identify prognostic features of crotonylation in HCC. Differential analysis, weighted gene co‐expression network analysis (WGCNA), and regression analysis were employed to construct a risk model. This study also evaluated the relationship between prognostic features, clinical characteristics and the immune microenvironment. Moreover, the regulatory mechanisms of these genes in HCC were explored. Finally, the expression of prognostic features was validated using reverse transcription‐quantitative polymerase chain reaction (RT‐qPCR). This research is pivotal in advancing the understanding of HCC pathogenesis.

## 2 MATERIALS AND METHODS

### 2.1 Data sources

In this study, high‐quality datasets from various public databases were utilized to ensure the reliability and broad applicability of the results. Initially, RNA‐seq data for hepatocellular carcinoma (LIHC) were sourced from The Cancer Genome Atlas (TCGA) database (http://cancergenome.nih.gov/), comprising 374 tumour samples and 50 healthy control samples. Clinical information and survival data for these samples were accessed via the UCSC Xena platform (https://xenabrowser.net/datapages/). For differential expression analysis, the hepatocellular carcinoma dataset GSE76427 was obtained from the Gene Expression Omnibus (GEO) database, consisting of 115 hepatocellular carcinoma tissue samples and 52 paraneoplastic control samples. Additionally, to externally validate the risk model and biomarker expression, 240 hepatocellular carcinoma cases (ICGC‐LIRI‐JP) were retrieved from the International Cancer Genome Consortium (ICGC) database (https://dcc.icgc.org). Furthermore, 18 lysine crotonylation‐related genes (LCRGs) were identified from a previous study.^16^

### 2.2 Differential analysis

Differential analysis was conducted on the TCGA‐LIHC and GSE76427 datasets to identify differentially expressed genes (DEGs) between tumours and controls. DESeq2 (v1.34.0)^17^ was employed to screen for DEGs (DEG1) in the TCGA‐LIHC dataset, while limma (v3.50.1)^18^ was used to identify DEGs (DEG2) in the GSE76427 dataset. The screening criteria were set at a *p*‐value <0.05 and |log_2_FC| > 0.5. Genes with consistent expression trends across both DEG1 and DEG2 were then overlapped to identify DEGs with uniform expression patterns across datasets.

### 2.3 Construction of WGCNA

The Wilcoxon test was used to assess the differential profiles of the 18 LCRGs in TCGA‐LIHC samples, and single‐sample gene set enrichment analysis (ssGSEA) was performed using GSVA (v1.42.0)^19^ to calculate the scores of differentially expressed LCRGs (DE‐LCRGs) among samples. These gene score profiles were then compared between tumour and control samples. To identify key module genes associated with DE‐LCRG scores, a co‐expression network was constructed using weighted gene co‐expression network analysis (WGCNA, v1.70.3).^20^ Clustering of all samples in TCGA‐LIHC samples was performed initially, with outliers being excluded. Optimal soft thresholds (*β*) for constructing the scale‐free network were determined by selecting those with an *R* ^2^ value close to 0.85 and average connectivity near zero. A clustering tree diagram was generated by calculating neighbourhood and similarity measures, followed by module segmentation using the dynamic tree‐cutting algorithm (minModuleSize = 300, mergeCutHeight = 0.2). Pearson correlation coefficients were then calculated between DE‐LCRG scores and the identified modules. The module with the highest correlation coefficient was designated as the key module, and the genes within this module were identified as key module genes.

### 2.4 Enrichment analysis

Candidate genes were identified by intersecting uniformly expressed DEGs with key module genes. To further elucidate the functions and pathways associated with these candidate genes, enrichment analysis was conducted using clusterProfiler (v4.2.2),^21^ focusing on Gene Ontology (GO) and Kyoto Encyclopedia of Genes and Genomes (KEGG) pathways, with significance set at *p* < 0.05.

### 2.5 Construction and assessment of a risk model

The Cox proportional hazards model, a widely used semiparametric method in survival analysis, provides flexibility by not requiring specific assumptions about the survival distribution. This flexibility allows the model to be more adaptable, as it does not depend on knowing the exact form of the underlying risk function. The Cox model is particularly effective in handling censored data, where some individuals' survival times are not fully observed, as it avoids assumptions about the specific distribution of survival times. In this study, univariate Cox analysis, using the ‘survival’ package (v3.3–1),^22^ was applied to the TCGA‐LIHC dataset to identify survival‐related candidate genes (*p* < 0.05).

Subsequently, genes with *p* < 0.05 were tested for adherence to the proportional hazards (PH) assumption. Genes meeting the PH assumption were then subjected to least absolute shrinkage and selection operator (LASSO) analysis using glmnet (v4.1–2)^23^ to identify prognostic features and construct a risk model. LASSO operates by applying a penalty to the regression coefficients, shrinking some to zero, thus facilitating variable selection. Risk scores were then calculated by integrating the penalty coefficients with gene expression levels of the identified prognostic features. The final risk model was established, with the risk score calculated using the following formula:

$$\text{Risk score}=\sum_{i=1}^{n}\left(\text{coefi}\times Xi\right)$$

In this context, *X*i represents the relative expression of the ith prognostic gene *i*, while ‘*coefi*’ denotes the LASSO Cox coefficient associated with the ith prognostic gene *i*. Patients were subsequently classified into two risk groups based on the optimal risk score cutoff. The ROC curve is a graphical representation that plots the true positive rate (sensitivity) on the vertical axis against the false positive rate (1‐specificity) on the horizontal axis, based on a range of different cut‐off values or decision thresholds used in dichotomous classification. The area under the curve (AUC), which approaches 1 as diagnostic accuracy improves, was calculated to assess the model's sensitivity and specificity. The model's predictive accuracy over 1–3 years was further assessed by plotting ROC curves using the survival ROC package (v1.0.3).^24^ Patients were also stratified into different expression groups based on the expression levels of each prognostic gene and corresponding survival data, using the optimal cutoff value. Kaplan–Meier (K–M) survival curves were then generated with the ‘survminer’ package (v0.4.9)^25^ to highlight survival differences among the groups. Finally, the risk model's validity was tested using the ICGC‐LIRI‐JP dataset.

### 2.6 Clinical characteristic analysis

To explore the relationship between various clinical characteristics and risk scores, data from patients with LIHC were stratified based on factors such as age, T‐stage, and tumour stage. Differences in risk scores among these clinical subgroups were evaluated using the Wilcoxon test. Additionally, Kaplan–Meier survival curves, coupled with the log‐rank test, were employed to determine survival differences across risk groups categorized by each clinical characteristic.

### 2.7 Gene set enrichment analysis

To investigate the functions and pathways associated with differential genes across various risk groups, background gene sets c2.cp.kegg.v7.1.symbols.gmt and GOc5.all.v7.1.symbols.gmt were selected from MSigDB (http://gsea‐msigdb.org). Differential analyses were conducted to identify genes with significant expression differences among the risk groups, with log_2_FC calculated and ranked from highest to lowest. These differential genes were then subjected to Gene set enrichment analysis (GSEA) using ClusterProfiler, with significance defined as *p* < 0.05.

### 2.8 Tumour immunoassays

The impact of prognostic genes on the tumour immune microenvironment (TIME) in HCC was assessed by first performing ssGSEA to measure the abundance of 28 immune cell types in tumour samples. Differences in immune cell abundances between risk groups were analysed using the Wilcoxon test. Correlations between differentially abundant immune cells and prognostic characteristics were also examined. Additionally, variations in 13 immune checkpoints between risk groups were investigated, alongside differences in TIDE scores. Immunocycle‐related datasets from the TIP website were used to calculate ssGSEA scores for each function through Gene Set Variation Analysis (GSVA), allowing the assessment of differences in active processes of the cancer immune cycle across risk groups. Finally, to identify gene mutations specific to different risk groups, mutation data from TCGA‐LIHC were analysed using maftools (v2.14.0).^26^

### 2.9 Cluster analysis and GSVA

To elucidate the associations and distinctions among different cancer subtypes, tumour samples from TCGA‐LIHC were clustered using ConsensusClusterPlus (v1.62.0),^27^ with characterization based on cumulative distribution function (CDF) curves. Kaplan–Meier (K–M) survival curves were subsequently plotted to identify suitable subtype groupings for differential analysis. Principal component analysis (PCA) was then employed to assess the extent of differentiation among these subtypes. To further explore the mechanisms driving survival differences, enrichment analyses were conducted on subtypes with significant survival disparities. Scores for each gene set were calculated across samples using c2.cp.kegg.v2022.1.Hs.symbols.gmt as the background, aiming to pinpoint significant differences in gene sets between subgroups.

### 2.10 Drug sensitivity analysis and establishment of molecular network

To evaluate the sensitivity of patients in various risk groups to common antineoplastic drugs, half‐maximal inhibitory concentrations (IC_50_) for 138 drugs in TCGA‐LIHC tumour samples were calculated using pRRophetic (v0.5).^28^ Differences in IC_50_ values across risk groups were then analysed to determine drug sensitivity patterns. Additionally, the regulatory role of prognostic characteristics in HCC was investigated by identifying transcription factors (TFs) from the CHEA database and microRNAs (miRNAs) from the mirnet database that potentially regulate these characteristics. Regulatory factors with a node count greater than 2 were selected to construct a TF‐mRNA‐miRNA network, providing deeper insights into the regulatory mechanisms influencing HCC.

### 2.11 Expression analysis of prognostic characteristics

The expression of prognostic characteristics was analysed in tumour and control samples from the TCGA‐LIHC and ICGC‐LIRI‐JP datasets. These findings were further validated through RT‐qPCR. For this purpose, 10 pairs of normal and HCC samples were collected from the First Affiliated Hospital of Harbin Medical University. Informed consent was obtained from all participants, and the study received ethical approval from the hospital's ethics committee (201909).

Total RNA was extracted from the samples using TRIzol reagent (Invitrogen, China), following the manufacturer's instructions. RNA concentrations were then measured using the NanoPhotometer N50. Complementary DNA (cDNA) was synthesized through reverse transcription using the SureScript First‐strand cDNA Synthesis Kit (Servicebio, China). Quantitative PCR (qPCR) was subsequently conducted with the CFX Connect Thermal Cycler (Bio‐Rad, USA), and relative mRNA quantification was achieved using the 2^−ΔΔCT^ method. Primer sequences are detailed in Table S1.

### 2.12 Statistical analysis

All statistical analyses were performed using R (v4.1.0), with *p* < 0.05 considered as the threshold for statistical significance.

## 3 RESULTS

### 3.1 Identification of 1179 uniformly expressed DEGs

**FIGURE 1:**
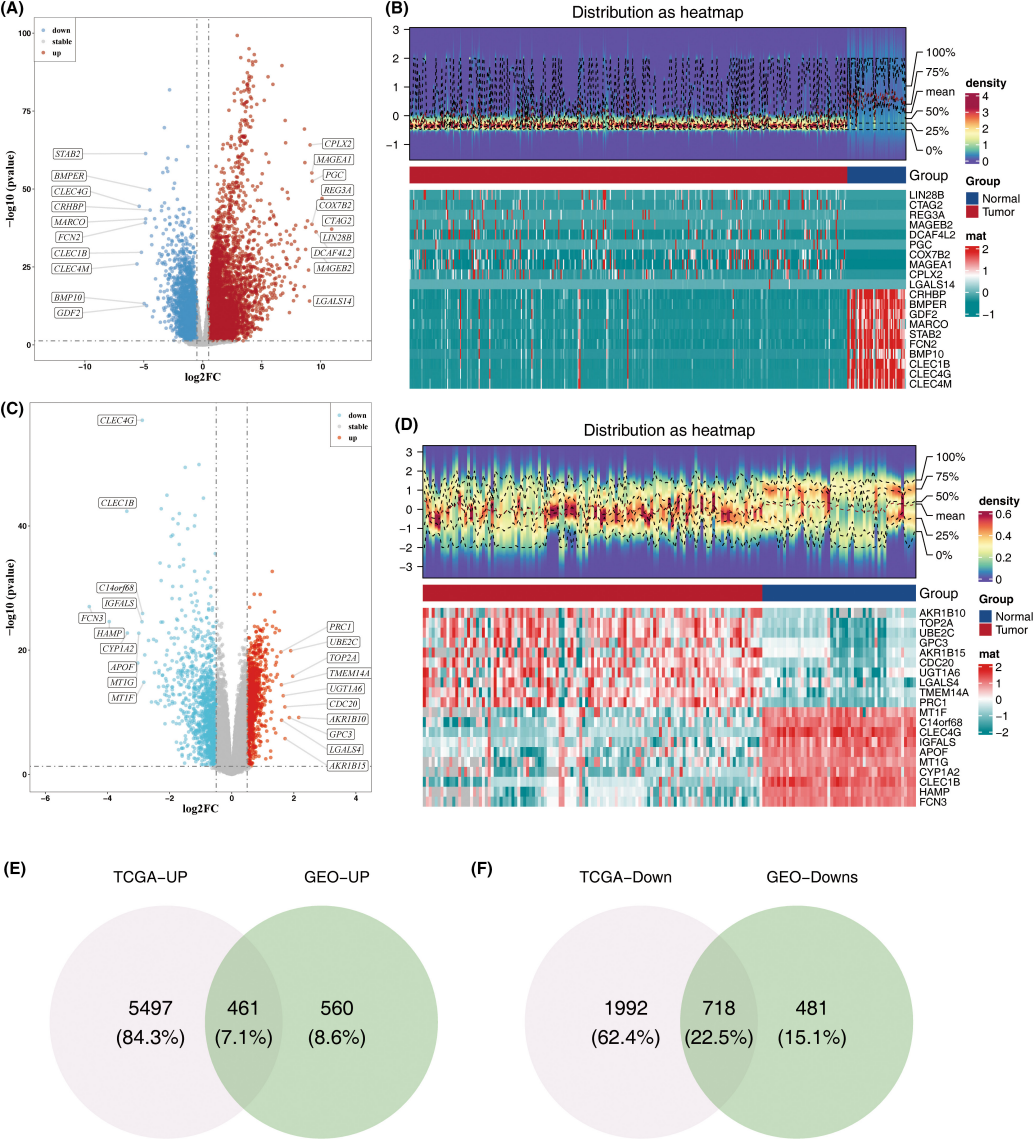
Identification of DEGs with consistent expression patterns. (A) Volcano plot of DEG1 in the TCGA‐LIHC dataset. (B) Heatmap showing the top 10 upregulated and downregulated DEG1. (C) Volcano plot of DEG2 in the GSE76427 dataset. (D) Heatmap displaying the top 10 upregulated and downregulated DEG2. (E) Venn diagram illustrating the overlap of upregulated DEGs. (F) Venn diagram illustrating the overlap of downregulated DEGs.

A total of 8668 DEG1s were identified, consisting of 2710 downregulated and 5958 upregulated genes, alongside 2220 DEG2s, which included 1199 low‐expressed and 1021 highly expressed genes. These DEGs were observed between tumour and control samples in the TCGA‐LIHC and GSE76427 datasets, respectively (Figure 1A–D). From these, 1179 DEGs with consistent expression patterns were identified, comprising 461 commonly upregulated and 718 commonly downregulated genes (Figure 1E,F).

### 3.2 Identification of 4200 key module genes via WGCNA

**FIGURE 2:**
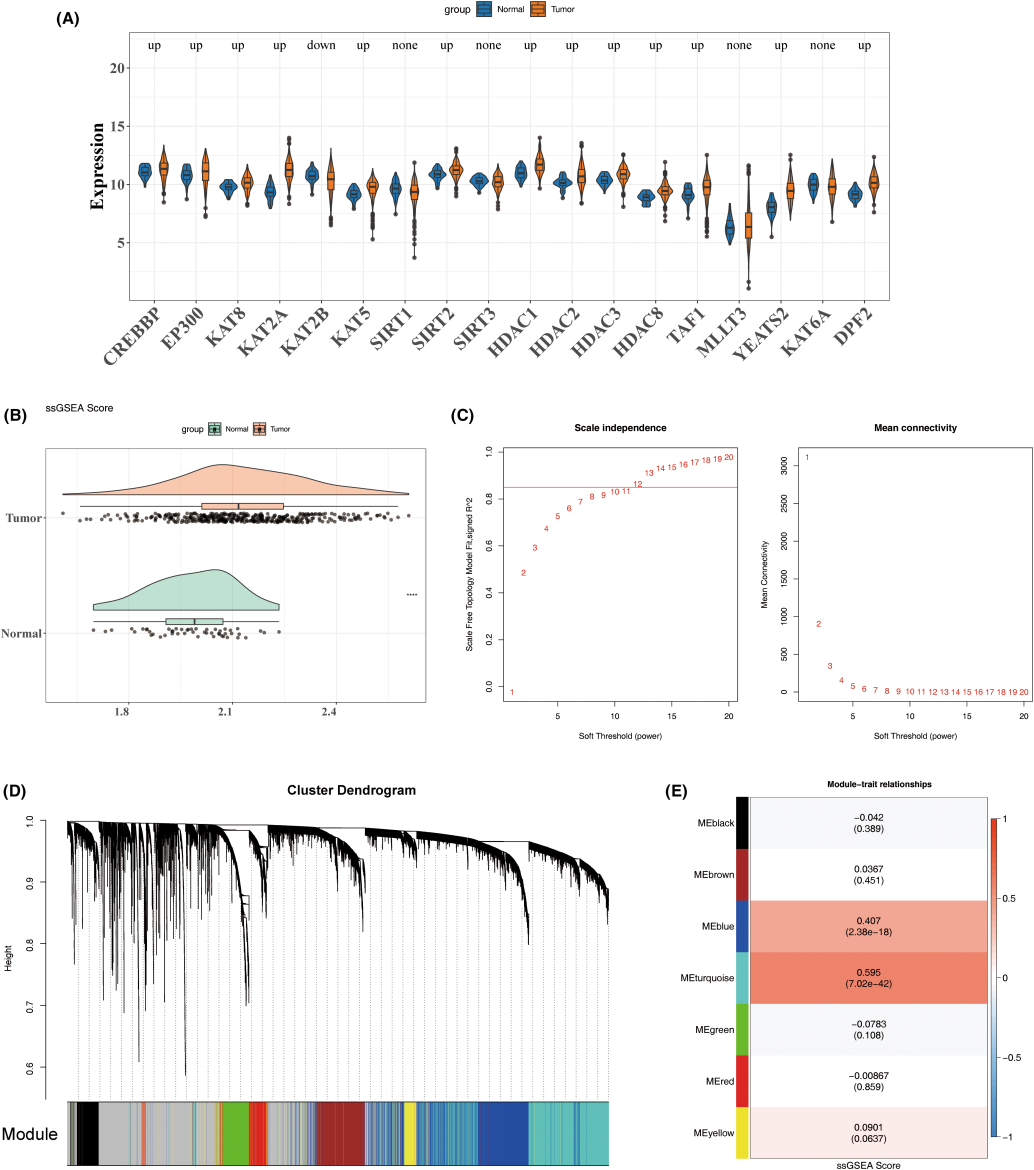
Identification of key module genes using WGCNA. (A) Differential analysis of LCRGs between tumour and control samples. (B) Differential analysis of ssGSEA scores between tumour and control samples. (C) Determination of the optimal soft‐thresholding power. (D) Identification of gene modules, with different colours representing distinct modules. (E) Heatmap illustrating the correlation between gene modules and ssGSEA scores.

The Wilcoxon test revealed 14 LCRGs with significant differences between tumour and control groups, which were selected for subsequent analyses (Figure 2A). The LCRG scores also exhibited similar distinctions between the groups, suggesting a correlation with the disease (Figure 2B). Cluster analysis confirmed the absence of outlier samples (Figure S1). A soft threshold of 12 was determined to be optimal for constructing a scale‐free network (Figure 2C), leading to the identification of seven gene modules (Figure 2D). Among these, the turquoise module, which contained 4200 genes, showed the strongest correlation with LCRG scores and was designated as the key module (Figure 2E).

### 3.3 Identification and functional enrichment of 88 candidate genes

**FIGURE 3:**
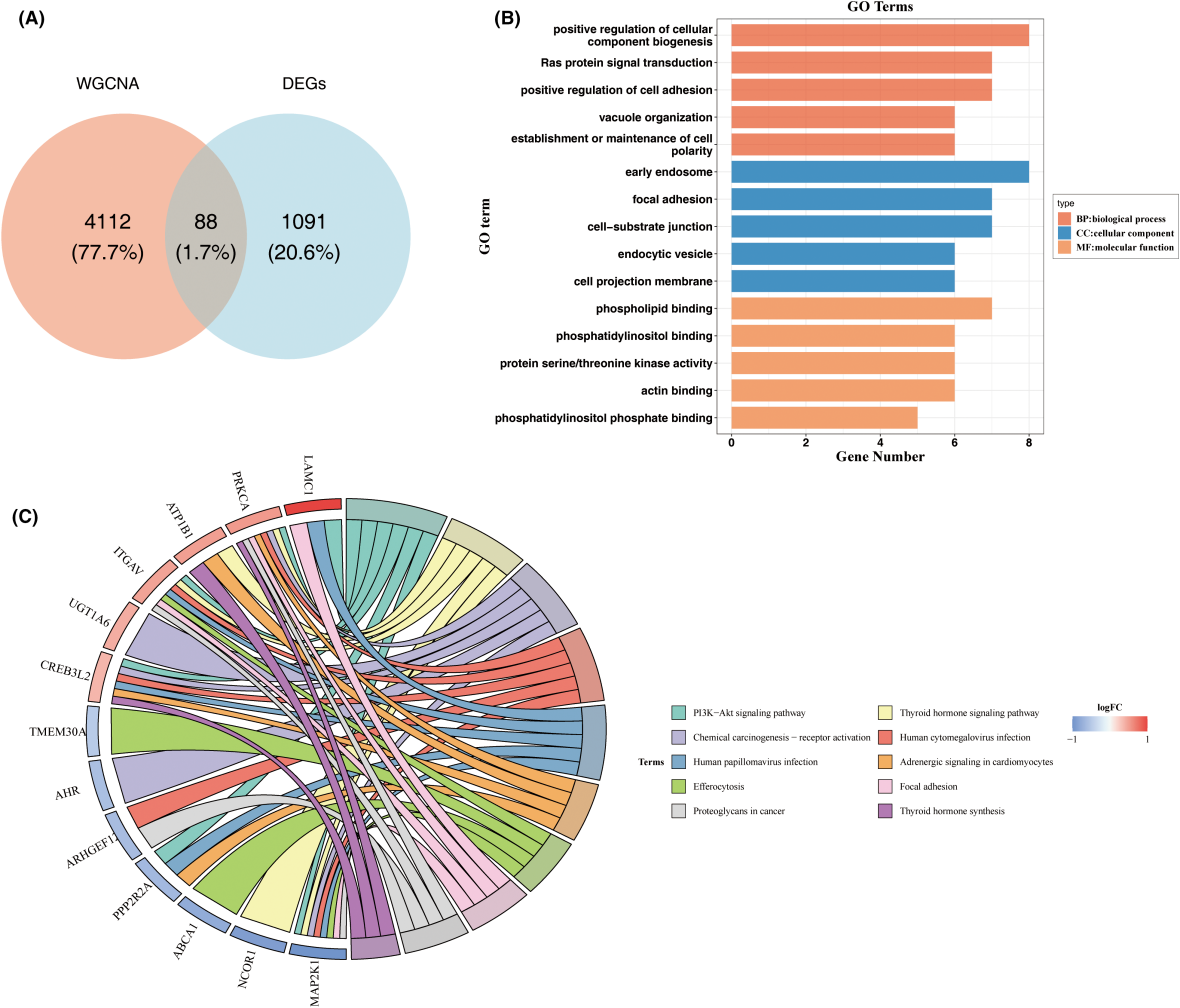
Identification and enrichment analysis of candidate genes. (A) Venn diagrams displaying the candidate genes. Enrichment analysis of candidate genes, including GO analysis (B) and KEGG analysis (C).

By intersecting the uniformly expressed DEGs with the key module genes, 88 candidate genes were identified (Figure 3A). GO analysis indicated that these genes were primarily involved in the positive regulation of cellular component biogenesis, early endosome processes, phospholipid binding, and Ras protein signal transduction (Figure 3B). KEGG pathway analysis further revealed enrichment in adrenergic signalling in cardiomyocytes, the PI3K‐AKT signalling pathway, proteoglycans in cancer, and chemical carcinogenesis‐receptor activation (Figure 3C).

### 3.4 Construction of a risk model based on seven prognostic characteristics

**FIGURE 4:**
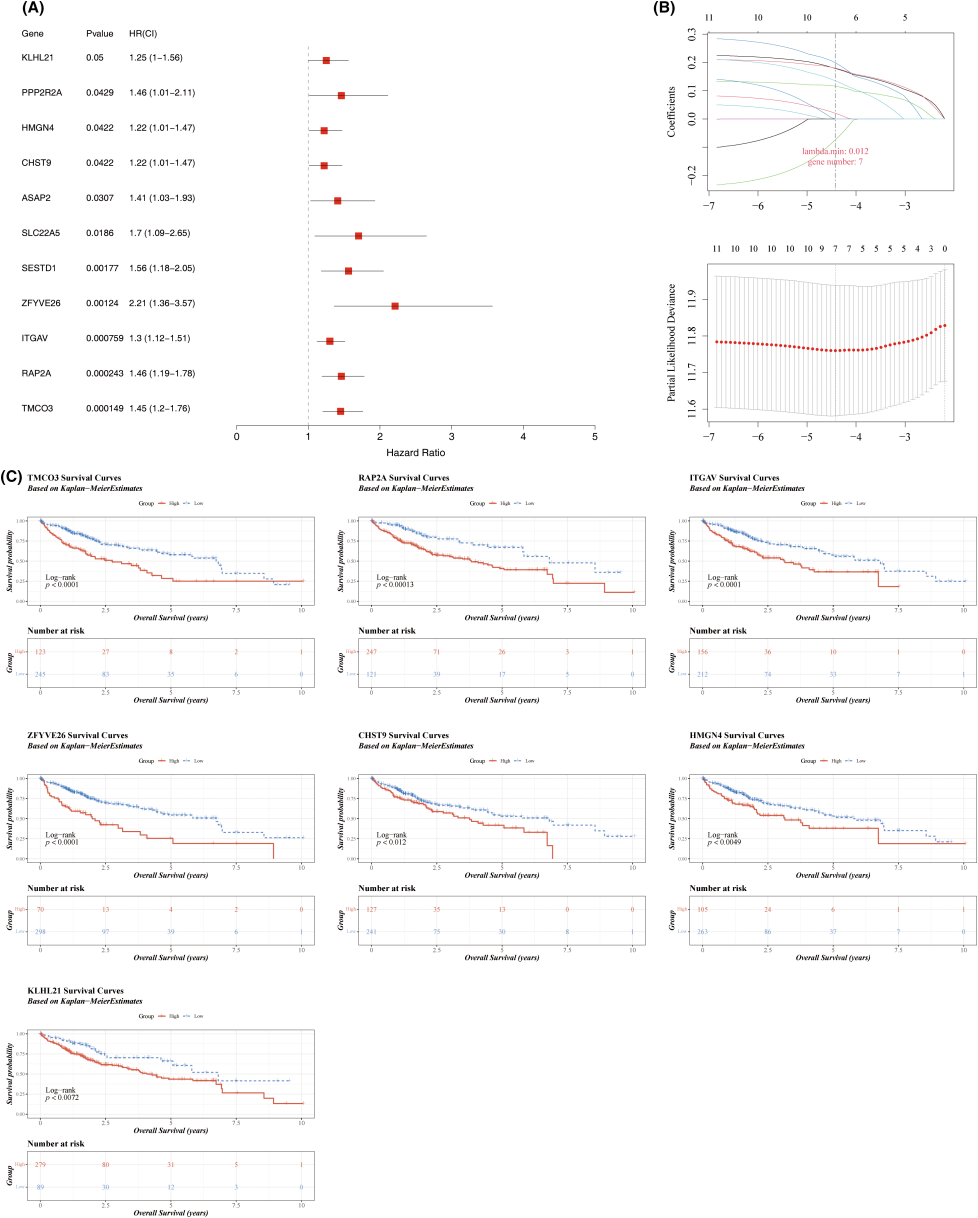
Construction of a risk model. (A) Forest plot of univariate Cox regression analysis. (B) LASSO regression analysis of prognostic features. (C) Survival curve analysis of prognostic features, with *p* < 0.05 indicating statistical significance.

To identify prognostic genes for HCC, a series of regression analyses were conducted on these 88 candidate genes. Initially, 11 survival‐related genes were identified, all of which passed the PH assumption test (Figure 4A). Subsequently, seven prognostic markers—transmembrane and coiled‐coil domain 3 (TMCO3), RAP2A, integrin subunit alpha‐V (ITGAV), ZFYVE26, CHST9, HMGN4, and Kelch‐like protein 21 (KLHL21)—were selected through 10‐fold cross‐validation using the optimal model parameter *λ* = 0.01201314, and a risk model was developed (Figure 4B). The risk score was calculated as follows: RiskScore = TMCO3×(0.179) + RAP2A×(0.18) + ITGAV×(0.116) + ZFYVE26×(0.199) + CHST9×(0.02) + HMGN4×(−0.073) + KLHL21×(0.135). Patients from the TCGA‐LIHC dataset were then stratified into risk groups based on the optimal cutoff value (−2.219387). Furthermore, patients were grouped according to the expression levels of each prognostic marker and their corresponding survival data. Kaplan–Meier survival curves demonstrated a lower survival probability for patients in the high‐expression group (Figure 4C).

### 3.5 Excellent predictive ability of the HCC risk model and correlation with clinical characteristics

**FIGURE 5:**
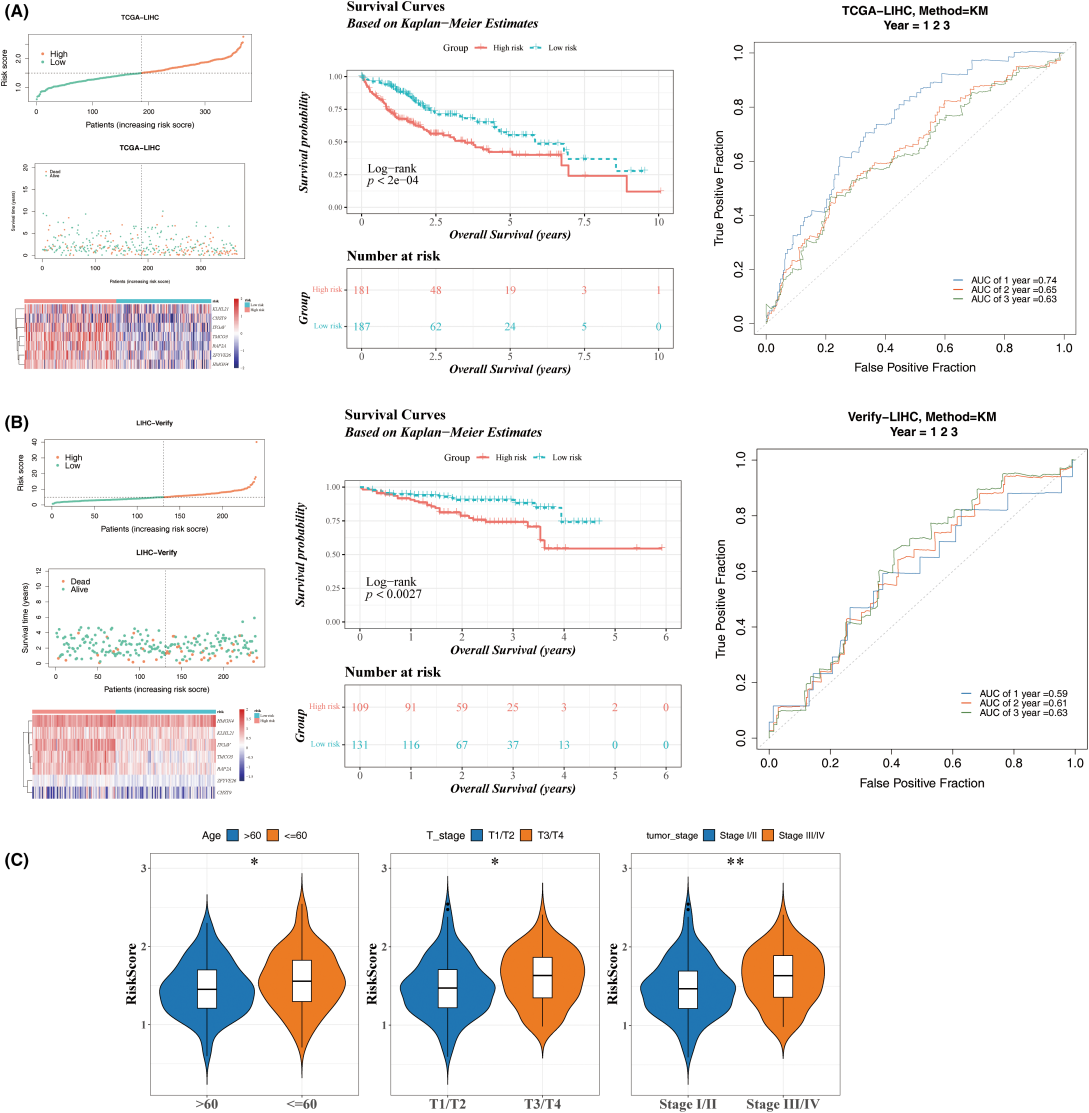
Assessment and validation of the risk model. (A) Risk score and overall survival (OS) distribution in the TCGA‐LIHC cohort, alongside the expression of prognostic characteristics in the high‐ and low‐risk groups (left). Survival curves for patients in the high‐ and low‐risk groups in TCGA‐LIHC (middle). The AUC for 1‐, 2‐, and 3‐year survival predictions in TCGA‐LIHC (right). (B) Risk score and OS distribution in the ICGC‐LIRI‐JP cohort, with the expression of prognostic characteristics in the high‐ and low‐risk groups (left). Survival curves for patients in the high‐ and low‐risk groups in the ICGC‐LIRI‐JP (middle). The AUC of ROC curves for 1‐, 2‐, and 3‐year survival predictions in ICGC‐LIRI‐JP (right). (C) Violin plots illustrating the differences in risk scores across age, T‐stage, and tumour stage. *p < 0.05; **p < 0.01.

To evaluate the model's reliability, survival analysis and ROC curve assessments were conducted on the TCGA‐LIHC and ICGC‐LIRI‐JP datasets. Initial survival distribution plots in both datasets revealed a progressive increase in risk scores from left to right (Figure 5A,B left). Additionally, the expression of prognostic characteristics across distinct risk groups was illustrated separately for each dataset (Figure 5A,B left). Kaplan–Meier curves demonstrated significant differences among the risk groups, indicating that patients in the high‐risk group had a markedly reduced probability of survival (Figure 5A,B middle). The AUC for 1‐ to 3‐year survival exceeded 0.63 in TCGA‐LIHC and was approximately 0.6 in ICGC‐LIRI‐JP (Figure 5A,B right), collectively underscoring the model's strong predictive capability for HCC. To further elucidate the relationship between Risk Scores and clinical features, notably elevated risk scores were observed in individuals aged ≤60 and those with T3/T4 stage tumours or III/IV tumour stages (Figure 5C). Additionally, K–M curves indicated significantly lower survival rates among patients over 60 in the high‐risk group (Figure S2).

### 3.6 Prognostic characteristics are involved in complex functions and pathways

**FIGURE 6:**
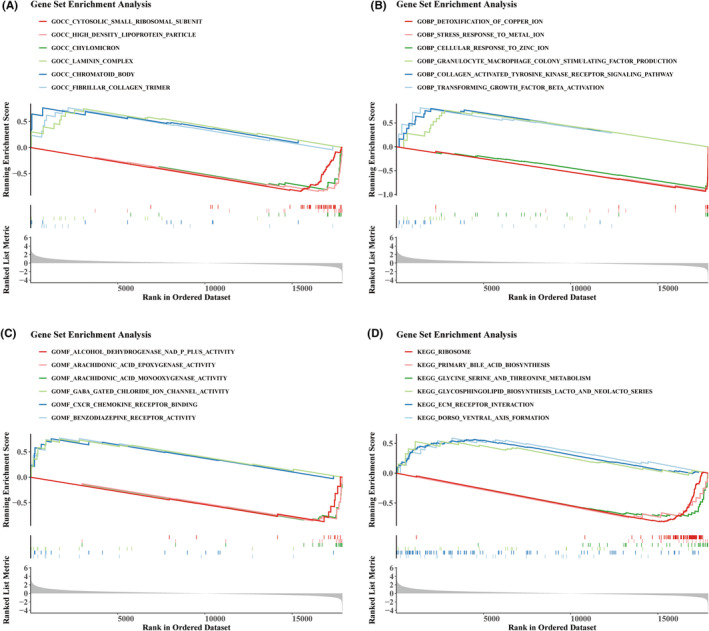
GSEA. (A) Enrichment of genes in the CC pathway. (B) Enrichment of genes in the BF pathway. (C) Enrichment of genes in the MF pathway. (D) Enrichment of genes in the KEGG pathway. The coloured curves on the graphs correspond to different functional pathways, with the vertical axis representing enrichment scores. The vertical lines within the graphs indicate the genes enriched in each pathway. The grey area below shows the horizontal axis representing genes sorted by logFC, while the vertical axis reflects the mapping of logFC.

To elucidate the pathways and functions associated with the prognostic characteristics, GSEA was performed. Cellular Component (CC)‐GO analysis revealed that these genes were primarily involved in structures such as fibrillar collagen, chromatoid bodies, laminin complexes, chylomicrons, high‐density lipoprotein particles, and cytosolic small ribosomal subunits. Biological Process (BP)‐GO analysis demonstrated enrichment in pathways related to transforming growth factor beta activation, collagen‐activated tyrosine kinase receptor signalling, granulocyte‐macrophage colony‐stimulating factor production, cellular responses to zinc ions, metal ion stress responses, and copper ion detoxification. Molecular Function (MF)‐GO analysis indicated participation in CXCR chemokine receptor binding, benzodiazepine receptor activity, GABA‐gated chloride ion channel activity, arachidonic acid epoxygenase activity, and alcohol dehydrogenase NAD(P) + activity (Figure 6A–C). Additionally, KEGG pathway analysis linked these genes to glycosphingolipid biosynthesis (lacto and neolacto series) and dorsoventral axis formation (Figure 6D). Collectively, these results suggest that these genes significantly influence the development of HCC through their involvement in a diverse array of functions and pathways.

### 3.7 Certain correlations of risk score with TIME

**FIGURE 7:**
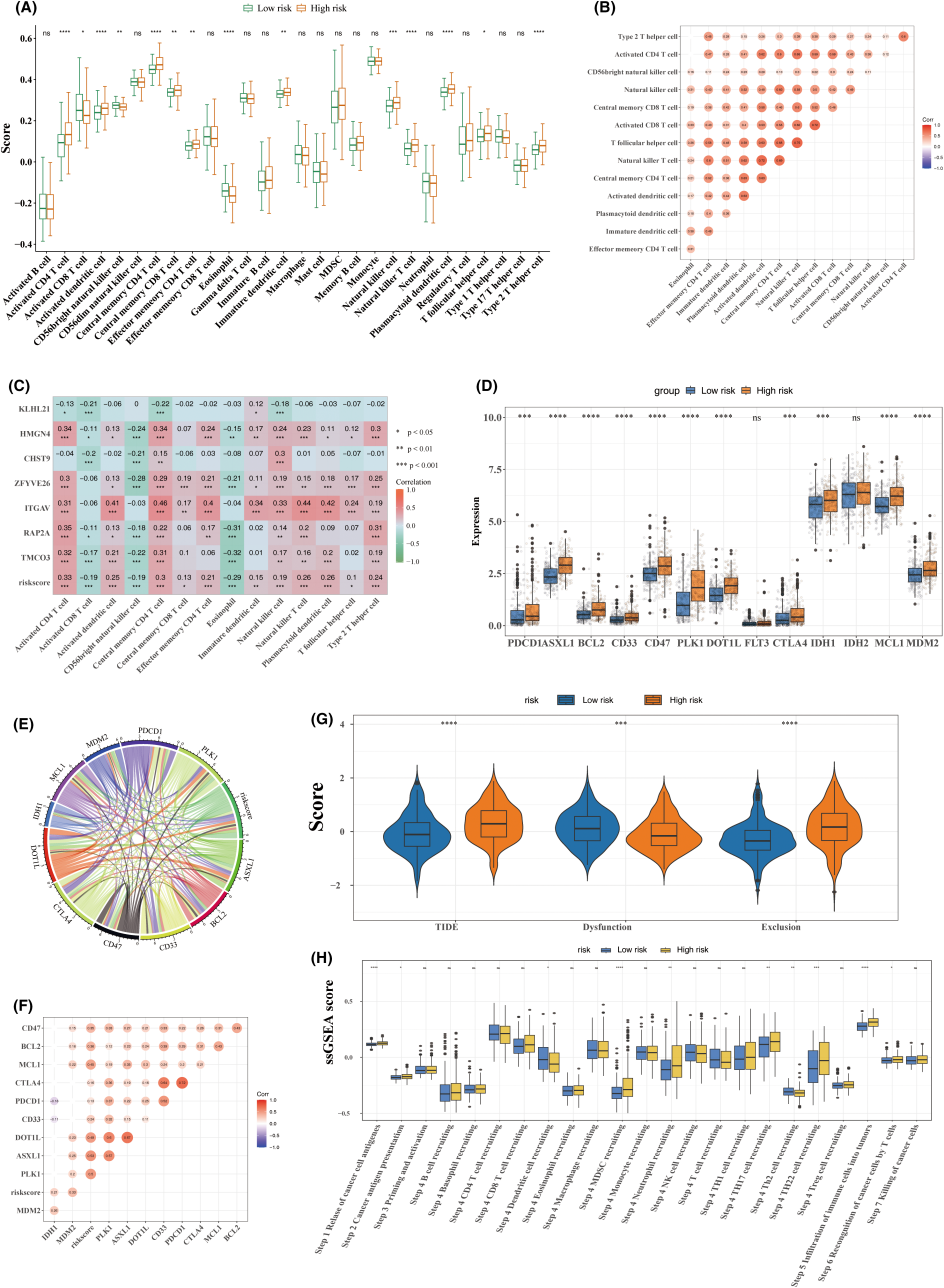
Analysis of the correlation between prognostic characteristics and the immune microenvironment. (A) Infiltration scores of 28 immune cell types in both high‐ and low‐risk groups. (B) Heatmap showing the correlations among differential immune cells. (C) Heatmap illustrating the relationships between risk scores, prognostic characteristics, and differential immune cells. (D) Expression levels of immune checkpoints in high‐ and low‐risk groups. (E, F) Correlation analysis between risk scores and differential immune checkpoints, with red indicating positive correlations and blue representing negative correlations; deeper colours signify stronger correlations. (G) Violin plot demonstrating differences in TIDE, dysfunction, and exclusion scores between high‐ and low‐risk groups. (H) Comparative analysis of immunological circulation between high‐ and low‐risk groups. *p < 0.05; **p < 0.01; ***p < 0.001; ****p < 0.0001.

The role of prognostic characteristics within the TIME of HCC was further investigated. Significant variations in immune cell types were observed between risk groups. Notably, except for CD56bright natural killer cells, eosinophils, and activated CD8+ T cells, which were significantly elevated in the low‐risk group, the remaining 11 immune cell types exhibited markedly higher levels in the high‐risk group, including activated CD4+ T cells, T follicular helper cells, and type 2 T helper cells (Figure 7A). Additionally, these differential immune cells showed positive correlations with one another (Figure 7B). Risk scores demonstrated a prominent correlation with these differential immune cells across all cases, with the strongest positive correlation observed with activated CD4+ T cells and the strongest negative correlation with eosinophils (Figure 7C). Scatter plots illustrating the relationship between risk scores and differential immune cells are provided in Figure S3. Furthermore, 11 immune checkpoints exhibited significant differences between risk groups (Figure 7D), with a notable positive correlation between CTLA4 and PDCD1 (Figure 7E). Risk scores also showed a strong correlation with ASXL1 (Figure 7F). The TIDE and exclusion scores were significantly higher in the high‐risk group, while the dysfunction score was lower (Figure 7G). Immune cycle processes related to Steps 1, 4, 5, and 6 were notably more pronounced in the high‐risk group (Figure 7H).

### 3.8 ZFYVE26 exhibited the highest mutation frequency in risk groups

**FIGURE 8:**
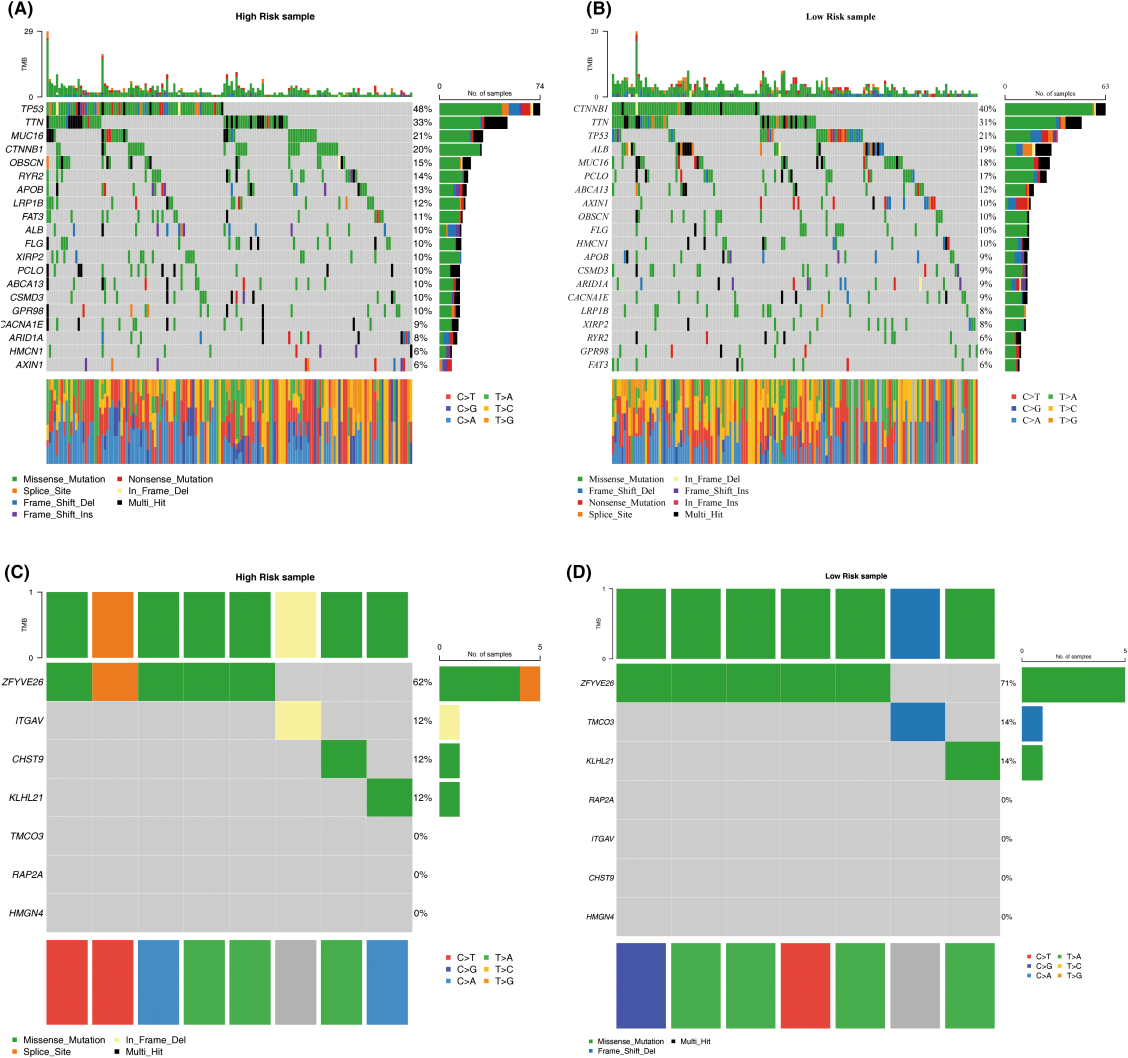
Mutation analysis of prognostic characteristics in high‐ and low‐risk groups in TCGA‐LIHC. (A, B) Mutation landscape of the top 20 mutated genes in the high‐ and low‐risk groups. (C, D) Mutation landscape of prognostic characteristic genes in the high‐ and low‐risk groups. Different colours denote various mutation types.

Mutation profiles across the different risk groups were assessed, revealing that TP53 had the highest mutation frequency (48%) in the high‐risk group, while CTNNB1 had the highest mutation frequency (40%) in the low‐risk group. Missense mutations emerged as the most prevalent mutation type across all risk groups (Figure 8A,B). Among the prognostic genes, ZFYVE26 exhibited the highest mutation frequency across the various groups (Figure 8C,D).

### 3.9 DEGs between subtypes are associated with multiple pathways

**FIGURE 9:**
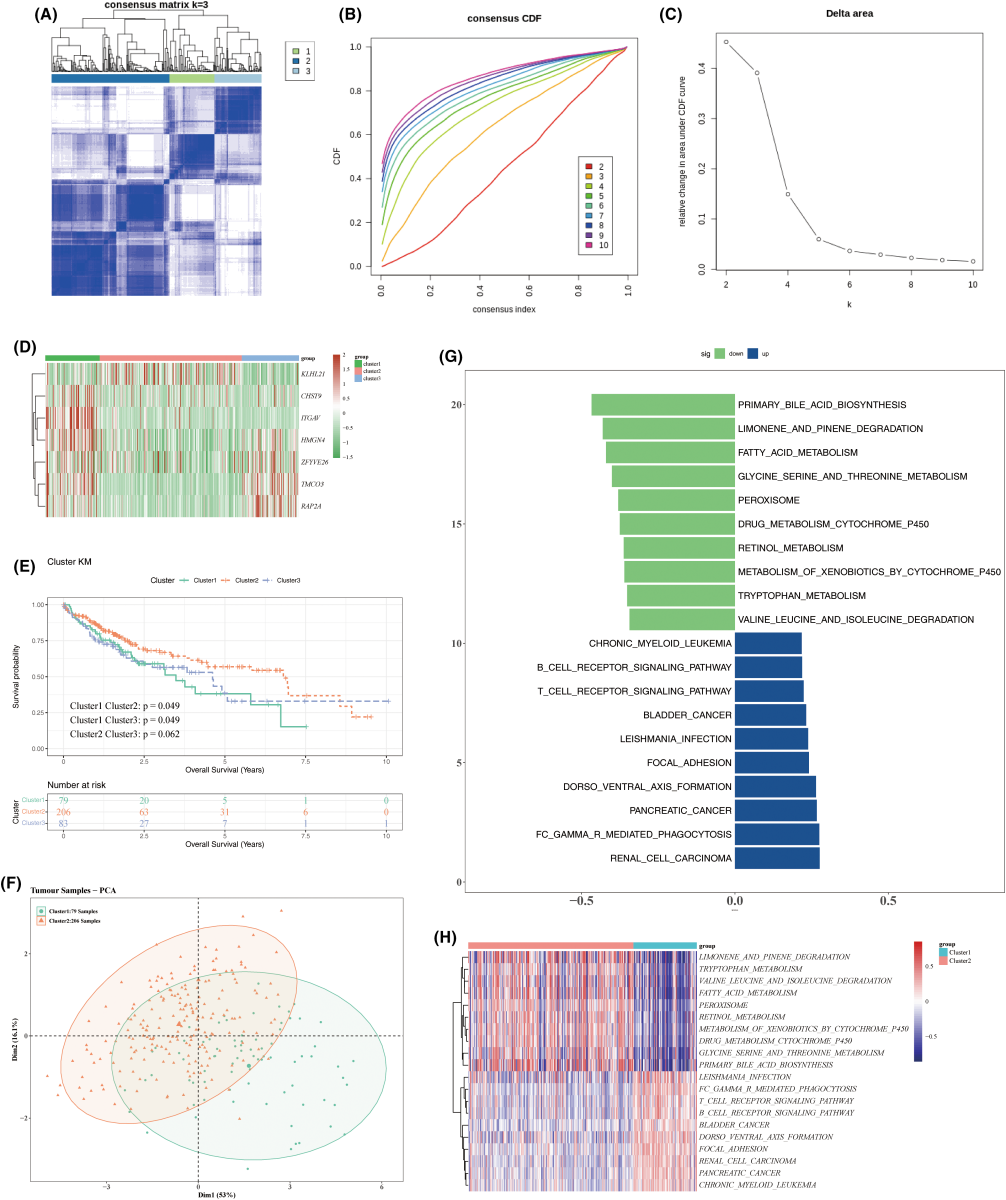
Subtype clustering of HCC tumour samples and pathway analysis. (A) Heatmap illustrating the clustering of tumour samples, with different colours on the horizontal axis representing distinct subtypes. (B, C) Consensus clustering CDF curve, showing consensus values ranging from 0 to 1. The relative change in area under the CDF curves as the number of clusters changes from *k* to *k* + 1, with *k* ranging from 2 to 10 and the optimal k determined to be 3. (D) Expression levels of prognostic genes across different subtype subgroups, with the horizontal axis representing samples from the three subtypes and the vertical axis showing prognostic gene expression. (E) K–M curves comparing survival rates across subtypes; the horizontal axis represents survival time, the vertical axis shows survival rates, and different coloured curves indicate the survival status of the subtype clusters. A *p*‐value <0.05 suggests a significant difference in survival between clusters. (F) PCA of Cluster 1 and Cluster 2, with different colours indicating subtype clusters, and areas of overlap suggesting some correlation between groups. (G) GSVA enrichment analysis bar graph, with green bars indicating the top 10 downregulated pathways and blue bars indicating the top 10 upregulated pathways. (H) Heatmap of GSVA enrichment analysis, where red represents relatively high expression and blue represents relatively low expression.

Cluster analysis of TCGA‐LIHC tumour samples was performed (Figure 9A), resulting in the categorization of samples into three subtypes based on CDF curves: Clusters 1, 2, and 3 (Figure 9B,C). The expression of prognostic characteristics across these three clusters is illustrated in Figure 9D. Kaplan–Meier (K–M) curves indicated significant survival differences between Cluster 1 and Cluster 2 (*p* = 0.049), as well as between Cluster 1 and Cluster 3 (*p* = 0.049) (Figure 9E). Additionally, principal component analysis (PCA) revealed notable differentiation between Cluster 1 and Cluster 2 (Figure 9F), collectively suggesting that these clusters are suitable for further analyses.

The investigation into pathways associated with differential gene expression among the subtypes identified several significant findings. Specifically, pathways related to fatty acid metabolism, metabolism of xenobiotics by cytochrome P450, valine, leucine, and isoleucine degradation, and primary bile acid biosynthesis were suppressed. Conversely, pathways associated with renal cell carcinoma, chronic myeloid leukaemia, and Fc gamma receptor‐mediated phagocytosis were activated (Figure 9G,H).

### 3.10 Prognostic characteristics play a vital role in HCC

**FIGURE 10:**
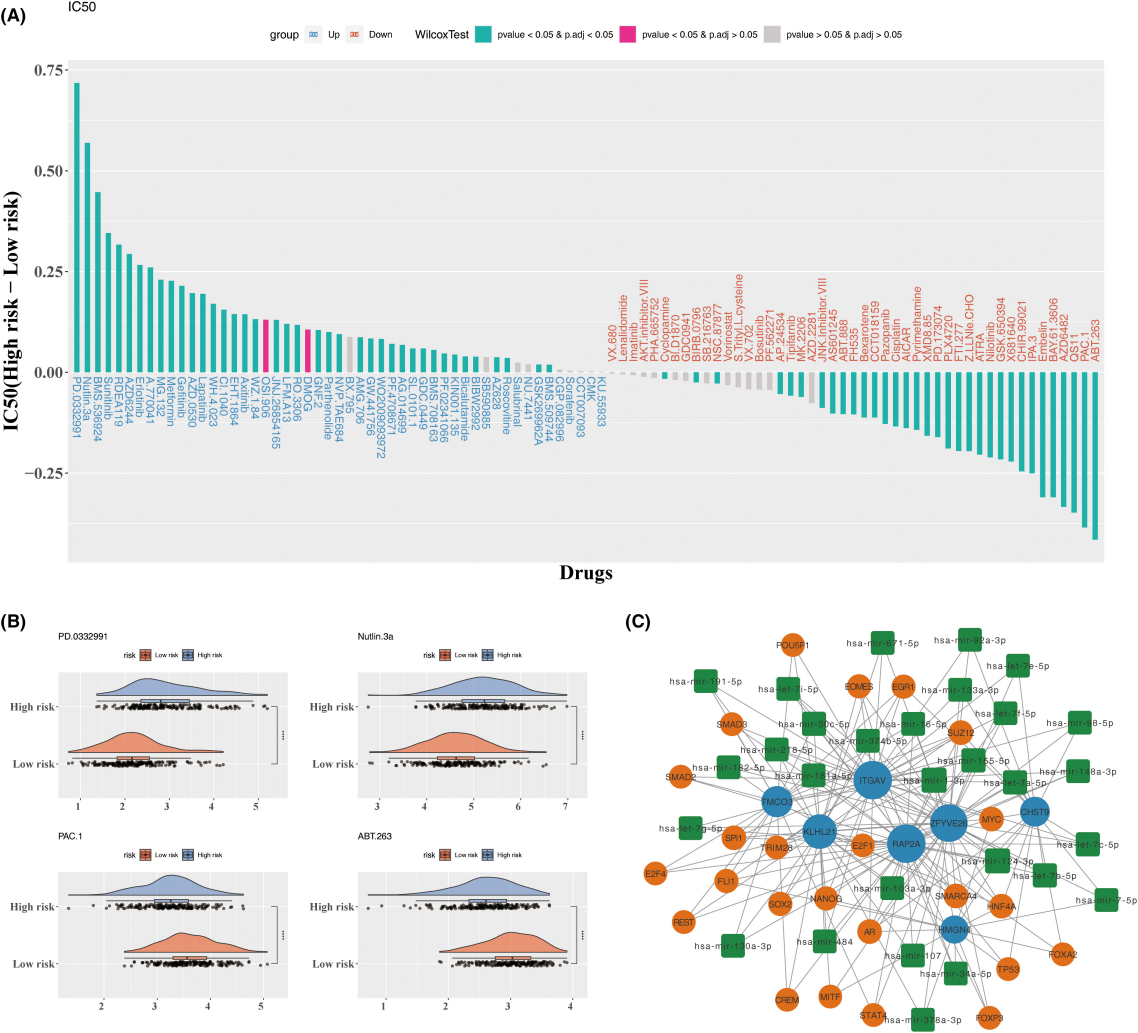
Drug sensitivity analysis and construction of a molecular regulatory network. (A) Differences in IC_50_ values of chemotherapeutic agents between the high‐ and low‐risk groups. (B) Cloud plot highlighting the four most significantly different drugs between the high‐ and low‐risk groups. (C) The TF‐mRNA‐miRNA regulatory network, with orange representing TFs, blue indicating prognostic characteristics, and green representing miRNAs.

The sensitivity of 138 drugs was further evaluated across risk groups, revealing significant differences in response among 75 drugs (Figure 10A). Notably, patients in the high‐risk group exhibited the greatest sensitivity to PD.0332991 and Nutlin.3a, while those in the low‐risk group showed increased sensitivity to PAC.1 and ABT.263 (Figure 10B). In addition, the regulatory mechanisms within HCC were explored by constructing a TF‐mRNA‐miRNA network, predicting the TFs and miRNAs associated with prognostic characteristics. Complex regulatory interactions were identified, such as SPI1‐TMCO3‐hsa‐mir‐130a‐3p and EOMES‐ITGAV‐hsa‐mir‐374b‐5p (Figure 10C).

### 3.11 Validation of the expression of prognostic characteristics

**FIGURE 11:**
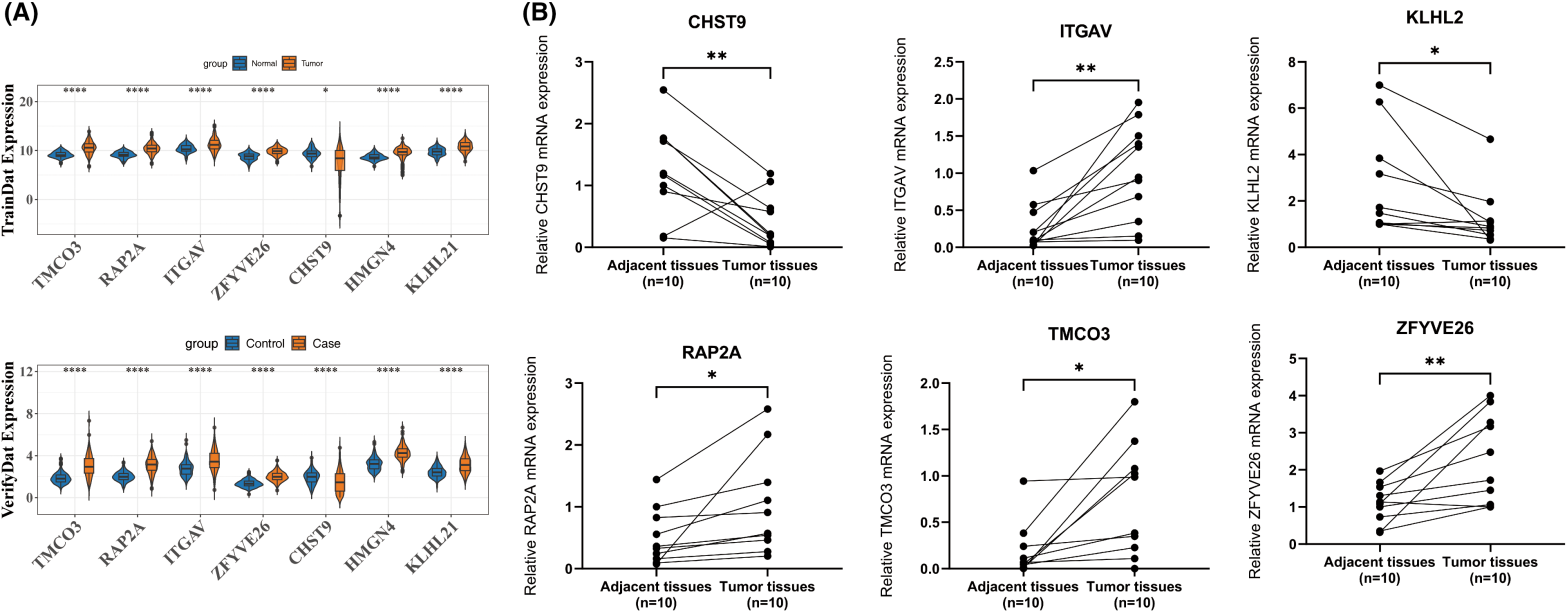
Validation of the expression of prognostic characteristics. (A) Expression levels of biomarkers across datasets, with brown representing the cancer group and blue representing the normal group. ****p < 0.0001. (B) RT‐qPCR expression analysis of biomarkers, where * indicates *p* < 0.05 and ** indicates *p* < 0.01.

All seven prognostic characteristics were prominently expressed in TCGA‐LIHC, with the exception of CHST9, which exhibited significantly lower expression levels in the tumour group. These genes demonstrated consistent and significant expression patterns in the ICGC‐LIRI‐JP dataset, mirroring those found in TCGA‐LIHC (Figure 11A). Additionally, RT‐qPCR analysis indicated that CHST9 was expressed at low levels in tumour samples, while HMGN4, ITGAV, RAP2A, TMCO3, and ZFYVE26 displayed high expression levels (Figure 11B).

## 4 DISCUSSION

HCC, primarily emerging from chronic liver disease, represents the most prevalent form of primary liver cancer. It is notably aggressive and its progression remains highly unpredictable.^29^ Although research has identified certain tumour markers, such as microvascular invasion (MVI) as a critical pathological feature of HCC, the findings are limited. The study in question elucidates the complex multicellular ecosystems associated with MVI and unveils distinct molecular characteristics, thereby offering a crucial scientific foundation for enhancing diagnostic accuracy and developing more effective therapies.^30^ Despite these insights, the availability of specific tumour markers is still constrained, leading to low early diagnosis rates and missed opportunities for partial hepatectomy and liver transplantation. Additionally, advanced stages of the disease remain inadequately addressed by arterial chemoembolization.^31^ The role of post‐translational modifications, particularly crotonylation, in liver cancer remains poorly understood. Studies indicate that elevated crotonylation levels in HCC facilitate cell invasion, contributing to recurrence and metastasis, and suggest its potential as an independent prognostic factor.^14^ Further exploration into crotonic acylation in other diseases reveals a strong correlation between crotonic acylation metabolic pathways and cancer progression, especially in relation to poor prognosis. Notably, lysine acetyltransferase 2A (KAT2A) expression is markedly increased in clear cell renal cell carcinoma (ccRCC), where it likely promotes tumour metastasis and proliferation by modulating the immune‐suppressive tumour microenvironment.^32^ This observation aligns closely with our findings. Consequently, a risk model for HCC was developed based on seven crotonylation‐related prognostic markers, providing a theoretical framework for both diagnosis and treatment.

This study investigates crotonic acylation‐related genes and their underlying molecular mechanisms in HCC through bioinformatics analysis. As an interdisciplinary field that integrates biology, statistics, and computer science, bioinformatics effectively processes and analyses large biological datasets. This approach not only enhances the speed of data processing but also enables comprehensive analysis from multiple dimensions. For instance, deep learning methods have proven highly effective in predicting metabolites associated with various diseases.^33^ An innovative study by Wang et al.^34^ employed a graphical attention mechanism and molecular fingerprinting, marking a new era in predicting drug‐induced liver injury. Meanwhile, the filter module full convolutional network (FM‐FCN) accurately extracts vital sign signals, while a novel filter within the multi‐engine evolution^35^ framework demonstrates excellent performance in diagnosing cardiovascular diseases. Recently, Zhu et al.^36^ introduced the Optimized Bee Algorithm (oBABC), which has achieved significant success in algorithm optimization. Additionally, the application of the DCAMCP model in cancer risk assessment has shown promising potential for drug design.^37^ The GAM‐MDR model effectively extracts microRNA‐drug interactions within networks of key nodes and pathways, significantly reducing the impact of noisy data.^38^ Recent studies indicate that graph autoencoders (GAE) excel in link prediction and node classification tasks. In particular, the FMSRT‐LPI model, which employs path masking and degree regression strategies, has yielded satisfactory results in predicting long noncoding RNA‐protein interactions (LPIs). These results highlight the broad applicability of bioinformatics. In summary, this study not only illustrates the role of bioinformatics in HCC research but also emphasizes its significance in personalized and precision medicine, offering new perspectives and tools for future drug design and disease treatment.

TMCO3, a key component of the molecular interaction network, plays a critical role in HCC tumour progression. This protein belongs to the monovalent cation proton antiporter‐2 family and functions as a Na^+^/H^+^ antiporter, regulating the ion gradient across the cell membrane.^39^ The upregulation of TMCO3 is linked to increased tumour growth and invasiveness, suggesting its involvement in the malignant transformation of HCC.^40^ Although the precise mechanism by which TMCO3 promotes tumour progression remains partially understood, evidence indicates that its activity is closely associated with the alteration of ion homeostasis in tumour cells.^41^ This relationship implies that TMCO3 may serve as a prognostic marker for patients with HCC, potentially guiding treatment decisions and monitoring disease progression. Future research will aim to elucidate the specific molecular mechanisms underlying TMCO3's oncogenic activity in HCC. Such insights will be vital for developing targeted therapeutic strategies designed to disrupt TMCO3's function, thereby reversing tumour progression and improving outcomes for patients suffering from this lethal disease.^40^

The role of the ITGAV gene in tumour progression has been extensively studied, particularly regarding its involvement in the invasion and metastasis of HCC.^42^ As a member of the integrin family, ITGAV is essential for cell adhesion and signalling, influencing cellular responses to extracellular matrix (ECM) components. In the context of HCC, activated ITGAV has been shown to enhance tumour invasion and metastasis by promoting the transcription of genes involved in oxidative phosphorylation, a vital process in cellular metabolism responsible for ATP production via the electron transport chain.^43^ Targeting ITGAV or its downstream signalling pathways may disrupt the invasive and metastatic potential of HCC cells, presenting a promising therapeutic strategy for this aggressive disease. Future research should aim to elucidate the specific mechanisms governing ITGAV‐mediated gene transcription and its effects on HCC progression. Such insights will facilitate the development of more effective treatment modalities for patients with this malignancy.^44^

KLHL21 has emerged as a significant player in the ubiquitin ligase pathway, profoundly influencing renal cell carcinoma progression. Its ability to facilitate the interpretation and ubiquitination of the ARHGEF7 protein provides a unique mechanism for inhibiting tumour growth in this context. However, the role of KLHL21 in HCC remains unclear, despite its importance in other cancers.^45^ Additionally, Kaplan–Meier survival curves indicated a low survival probability among patients in the high‐expression group, potentially due to uncontrollable factors such as sample variability or experimental conditions. Further validation through animal studies and Western blot analysis is essential to clarify these findings.

GSEA identified two pathways highly relevant to HCC, with SNHG16 significantly activating ECM‐receptor interaction, suggesting its involvement in cell adhesion and migration. This finding aligns with prior studies demonstrating that SNHG16 regulates cellular processes through interactions with ECM receptors. The substantial activation of ECM‐receptor interaction by SNHG16 indicates a critical role for long non‐coding RNA (lncRNA) in these cellular functions.^46^ Further investigations are needed to elucidate the mechanisms by which SNHG16 regulates ECM‐receptor interactions and its impact on cellular processes and disease progression.^46^ FIGNL1 has also been shown to promote the growth of tumour tissues and HCC cell lines by re‐establishing HMMR‐mediated ECM‐receptor interaction. This insight provides a novel perspective on the pathogenesis of HCC and highlights potential new targets for treatment. A series of comprehensive experiments conducted by researchers revealed a strong correlation between FIGNL1 expression and the migration, proliferation, and invasion capabilities of HCC cells. Silencing FIGNL1 in these cells significantly reduced their proliferative capacity and severely inhibited their migratory and invasive abilities, implicating FIGNL1 in HCC development and progression. These insights not only enhance the understanding of HCC pathogenesis but also suggest potential therapeutic targets.^47^ Future research will focus on exploring the specific mechanisms by which FIGNL1 contributes to liver cancer, offering new avenues for treatment.^47^ Research on HCC has also shown increased expression of enzymes involved in bile acid biosynthesis, leading to elevated bile acid levels. This upregulation is linked to oncogenic signalling pathways such as Wnt/β‐catenin and Hedgehog. The accumulation of bile acids exerts tumorigenic effects in HCC by promoting cell proliferation, inducing epigenetic alterations, and facilitating angiogenesis.^48^ Therefore, inhibiting bile acid biosynthesis is critical for HCC development and prognosis.

Bile acids activate oncogenic pathways that enhance cell proliferation and survival. Additionally, they induce epigenetic changes that contribute to the malignancy of HCC cells and promote angiogenesis through the activation of VEGF and other angiogenic factors. Targeting bile acid biosynthesis emerges as a promising therapeutic strategy for HCC. Inhibiting key enzymes in this pathway can lower harmful bile acid levels, thereby suppressing tumour growth and progression. Currently, drugs aimed at inhibiting bile acid biosynthesis are undergoing preclinical and clinical trials for HCC treatment, with studies ongoing to evaluate their efficacy and safety. The inhibition of bile acid biosynthesis is essential for HCC development and prognosis,^49^ and targeting this pathway offers a promising therapeutic approach for managing this aggressive disease.^50^ The synthesis of primary bile acids influences SMYD5, and silencing its expression may impede liver cancer progression,^51^ suggesting another potential therapeutic avenue.

Fourteen immune cell types exhibited significant differences between the risk groups, with the risk score showing the strongest positive correlation with activated CD4+ T cells and the strongest negative correlation with eosinophils. Chi Ma et al. demonstrated using both mouse models and human samples that dysregulation of lipid metabolism in nonalcoholic fatty liver disease leads to the selective depletion of activated CD4+ T cells within the liver, which accelerates hepatocarcinogenesis. In vivo inhibition of reactive oxygen species (ROS) effectively reversed the decrease in hepatic‐activated CD4+ T cells induced by nonalcoholic fatty liver disease, subsequently delaying the progression of HCC.^52^ Emerging evidence indicates that eosinophils infiltrate solid tumours and interact with tumour cells and lymphocytes through two primary mechanisms. First, eosinophils release cytokines and chemokines that modulate the tumour microenvironment, promoting angiogenesis, immune suppression, and tumour growth. Second, they can directly eliminate tumour cells by releasing cytotoxic granules containing enzymes such as major basic protein (MBP) and eosinophil peroxidase (EPO).^53^ These results have important implications for cancer immunotherapy, positioning eosinophils as potential targets for novel anticancer drugs due to their direct cytotoxic effects on tumour cells. Furthermore, the complex interactions between eosinophils, tumour cells, and lymphocytes highlight the potential of immune system modulation to enhance the antitumor immune response. Additionally, there is growing interest in harnessing the immune system to combat cancer, particularly by directing T cells toward tumour cells using chimeric antigen receptors (CARs). Given the role of eosinophils in tumour immunology, the prospect of genetically modifying them to express CARs may enable these cells to directly target and eliminate tumour cells. However, the exact role of eosinophils in tumour immunology remains incompletely understood.

Future research is essential to elucidate the mechanisms driving eosinophil infiltration in solid tumours and their interactions with tumour cells and lymphocytes. Such studies could lead to the development of innovative immunotherapeutic strategies that leverage the antitumor capabilities of eosinophils to enhance patient outcomes in cancer treatment.^53^ Members of the ASXL family exhibit context‐dependent oncogenic or tumour suppressor properties. Among them, ASXL1 plays a role in transcriptional repression through its interaction with PRC2 and is also involved in transcriptional regulation via interactions with BAP1 and/or the NHR complex. Truncation mutations in ASXL1 have been implicated in HCC.^54^

The establishment of the TF‐mRNA‐miRNA network enhances the understanding of interactions among TFs, mRNAs, and miRNAs within a specific biological context. This study identified significant TFs, such as SPI1 and SUZ12, along with the miRNAs hsa‐mir‐155‐5p and hsa‐mir‐124‐3p, which play vital roles in regulating gene expression and various cellular processes.

SPI1, also known as PU.1, is a member of the ETS family of TFs and is known to regulate haematopoiesis and stem cell differentiation. It interacts with both mRNAs and miRNAs, influencing their expression and functions. SUZ12, a component of PRC2, participates in epigenetic gene silencing, helping maintain cellular identity and repressing the transcription of specific genes. The miRNAs hsa‐mir‐155‐5p and hsa‐mir‐124‐3p post‐transcriptionally regulate gene expression and impact processes such as neuronal development, immune responses, and inflammation.^55^ The PRDM1‐USP22‐SPI1 axis regulates PD‐L1 levels, resulting in the depletion of infiltrating CD8+ T cells. Additionally, the overexpression of PRDM1, in conjunction with PD‐L1 monoclonal antibody therapy, represents a novel strategy for treating HCC.^56^ Furthermore, downregulation of SUZ12 can accelerate the invasion and metastasis of HCC cells, potentially mediated by SUZ12's inhibition of ERK1/2, MMP2, and MMP9 expression in these cells.^57^ The miRNA hsa‐mir‐124‐3p has been implicated in tumour progression in various malignancies, with decreased expression associated with shorter overall survival and poor prognosis in patients with HCC.^58^ The combined assessment of hsa‐mir‐155‐5p and AFP has demonstrated improved sensitivity in diagnosing HCC, and the ratio of hsa‐mir‐155‐5p to hsa‐mir‐199a‐5p has emerged as a promising molecular marker for patients with AFP‐negative HCC.^59^

Significant differences in IC_50_ values for 75 drugs were observed between the two risk groups, with the most notable differences associated with four drugs: PD.0332991, Nutlin.3a, PAC.1, and ABT.263, corresponding to the clinical agents palbociclib, rebemadli, procaspase‐activating compound‐1, and navitoclax, respectively. In human HCC cell lines, palbociclib inhibits cell proliferation by inducing reversible cell cycle arrest and, either alone or in combination with sorafenib, inhibits tumour growth in vivo while significantly prolonging survival.^60^ Rebemadli has been shown to enhance apoptosis mediated by MRPL21 deficiency in hepatoma cell lines Hep3B and HCCLM3 by inhibiting P53 activity.^61^ The proportion of platelet procaspase‐activating compound‐1+ serves as a novel indicator for diagnosing and predicting postoperative prognosis.^62^ Navitoclax increases the mRNA and protein levels of Mcl‐1 in hepatoma cells, contributing to resistance against the drug. Notably, Mcl‐1 mRNA levels can be elevated by enhancing mRNA stability without affecting transcription, and inhibitors of ERK, JNK, or AKT have been shown to enhance navitoclax‐induced apoptosis in HCC cells.^63^

This report represents the first identification of crotonylation‐associated prognostic characteristics in HCC. However, the study primarily relied on bioinformatics methods to explore the potential molecular mechanisms of genes associated with crotonylation in HCC. It is important to acknowledge the relatively small sample size and the inherent limitations of the analytical methods employed. Additionally, the underlying mechanisms were investigated solely at the gene expression level, which may not directly correlate with biological outcomes. Therefore, further animal and clinical studies are essential to validate these findings across various experimental settings. Furthermore, the clinical applicability of liver cancer‐specific tumour markers requires confirmation through larger sample sizes, and the use of targeted therapies necessitates additional clinical validation. Follow‐up experiments are planned to provide robust theoretical support for these findings.

## 5 CONCLUSION

This study confirmed that crotonylation facilitates HCC progression and that its elevated expression correlates with poorer prognosis in patients with HCC, underscoring its potential as a therapeutic target. The research provides a theoretical basis for phenotypic assays, including proliferation, scratch, migration, EDU, and Transwell experiments. To address the limitations of this study, future efforts will focus on validating these conclusions through larger‐scale animal experiments and clinical studies. Furthermore, we plan to conduct extensive validations across diverse patient populations, encompassing different ages, genders, races, disease stages, and other pertinent variables, to evaluate the accuracy and reliability of these predictive features. In parallel, the integration of these features into personalized treatment regimens will be explored, with the goal of offering patients more precise and effective therapeutic options. These subsequent studies are expected to yield a deeper understanding of the mechanisms involving legylation‐related genes in HCC, thereby laying a robust foundation for the advancement of personalized and precision medicine.

## AUTHOR CONTRIBUTIONS

**Bailu Yang:** Conceptualization (lead); data curation (equal); methodology (lead); project administration (lead); resources (equal); writing – original draft (lead). **Fukai Wen:** Data curation (lead). **Yifeng Cui:** Conceptualization (equal); funding acquisition (supporting); resources (equal); writing – review and editing (lead).

## FUNDING INFORMATION

The Heilongjiang Postdoctoral Science Foundation (Grant No. LBH‐Z20178). The Scientifc Foundation of the First Affiliated Hospital of Harbin Medical University (Grant No.2021B03). Excellent Youth Science Fund of the First Affiliated Hospital of Harbin Medical University (Grant No. 2021Y01).

## CONFLICT OF INTEREST STATEMENT

All authors declare no financial or personal relationships with any individual or organization that could inappropriately influence this work.

## Supporting information


**Figure S1.** Clustering of all samples.

**Figure S2.** Survival curves for different clinical subtypes among patients in high‐ and low‐risk groups.

**Figure S3.** Scatter plots showing the correlation between risk scores and differential immune cells.

**Table S1.** Primer sequences for prognostic characteristic genes.

## Data Availability

The data utilized in this study are accessible through the UCSC Xena (http://xena.ucsc.edu/), TCGA (http://cancergenome.nih.gov/, TCGA‐LIHC), GEO (https://www.ncbi.nlm.nih.gov/geo/, GSE76427), and ICGC (https://gwas.mrcieu.ac.uk/, ICGC‐LIRI‐JP) databases.
