# Supplementary figures and images for: Integrative transcriptome analysis identifies a crotonylation gene signature for predicting prognosis and drug sensitivity in hepatocellular carcinoma
Source: J Cell Mol Med. 2024 Oct 20;28(20):e70083. doi: 10.1111/jcmm.70083 (PMC11491312; doi:10.1111/jcmm.70083)

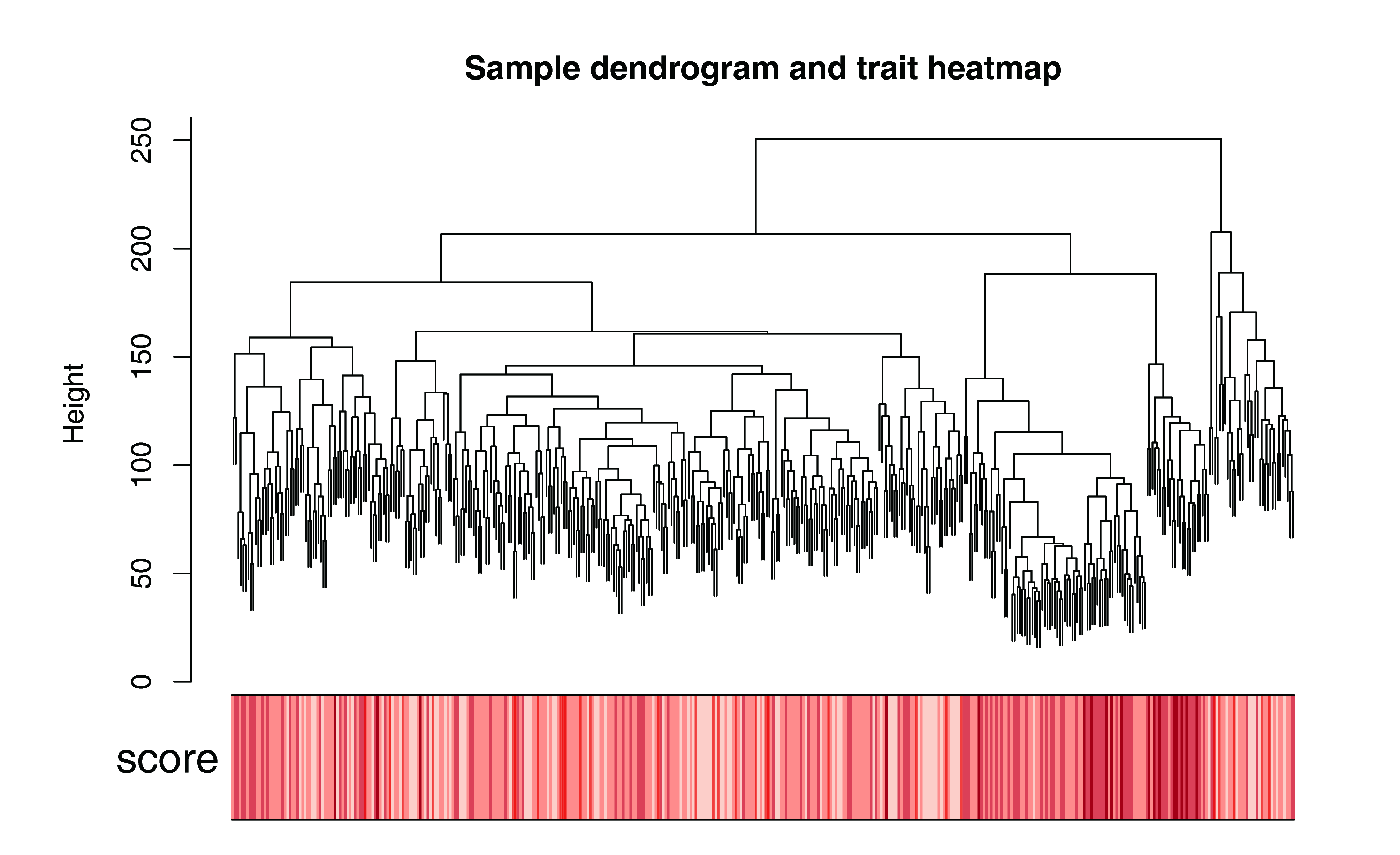

Supplement: Supplementary file 1 — Figure S1. Clustering of all samples. [file JCMM-28-e70083-s003.tif]

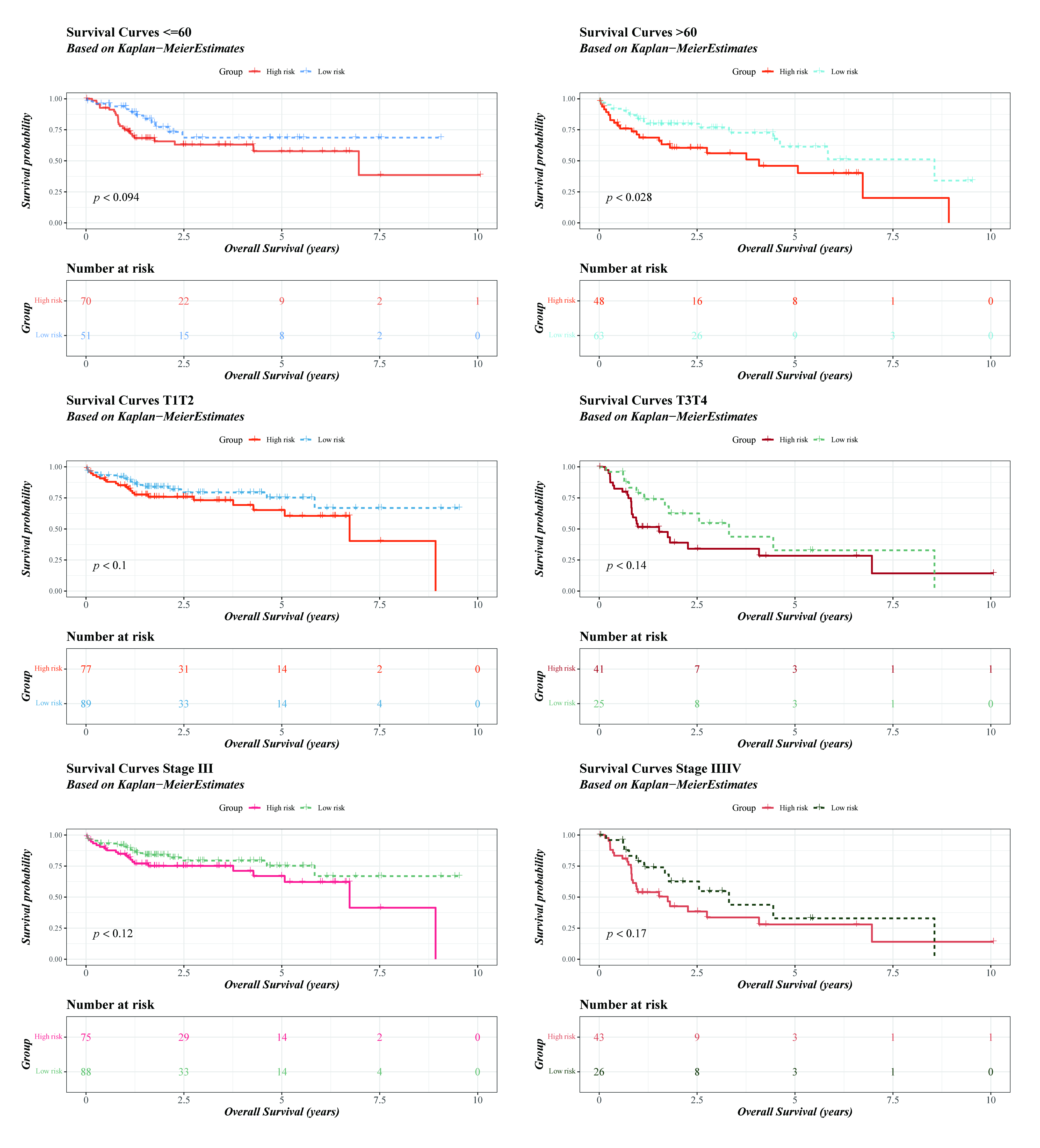

Supplement: Supplementary file 2 — Figure S2. Survival curves for different clinical subtypes among patients in high‐ and low‐risk groups. [file JCMM-28-e70083-s001.tif]

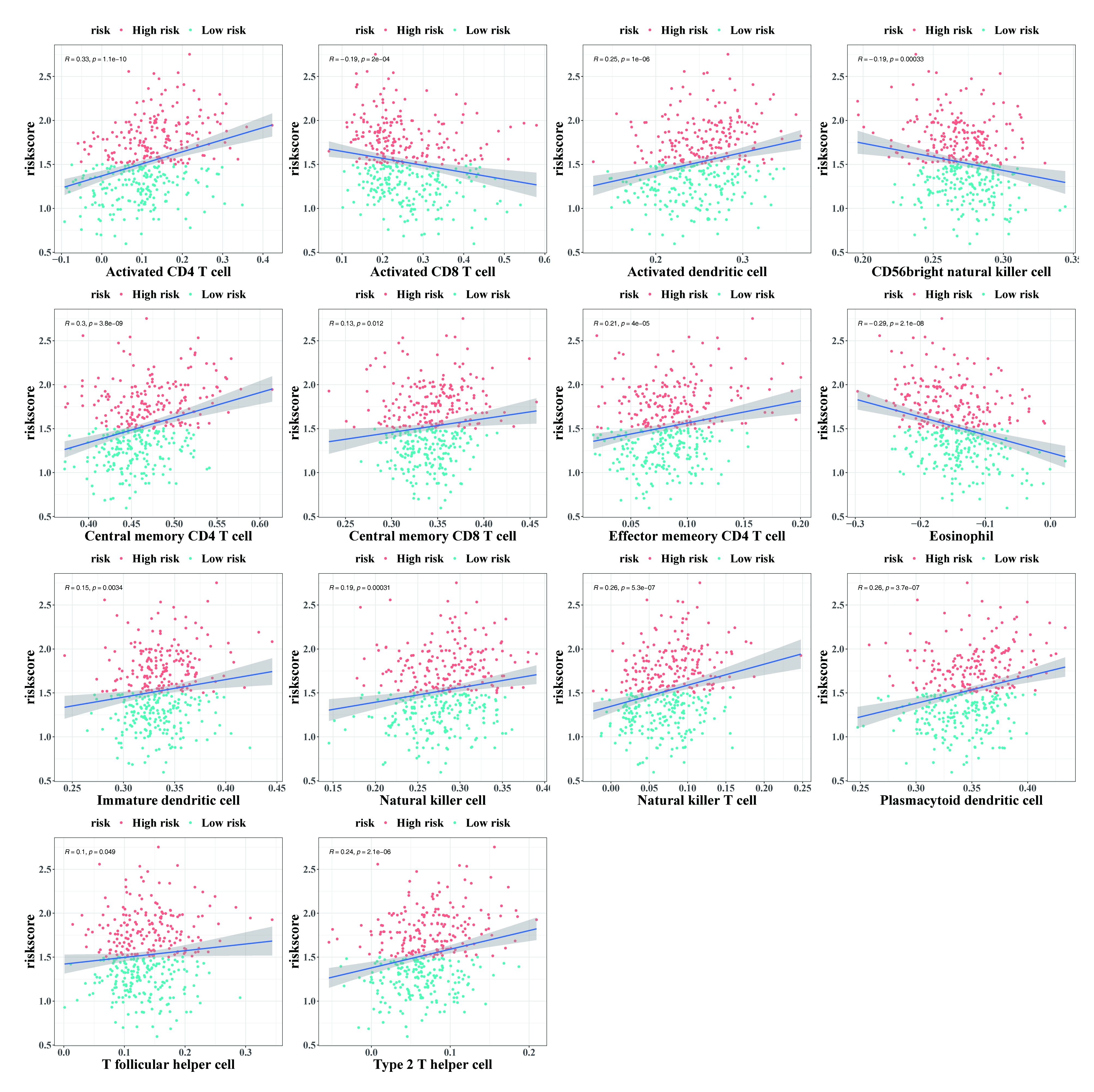

Supplement: Supplementary file 3 — Figure S3. Scatter plots showing the correlation between risk scores and differential immune cells. [file JCMM-28-e70083-s004.tif]
