# Supplementary material for: Integrative transcriptome analysis identifies a crotonylation gene signature for predicting prognosis and drug sensitivity in hepatocellular carcinoma
Source: J Cell Mol Med. 2024 Oct 20;28(20):e70083. doi: 10.1111/jcmm.70083 (PMC11491312; doi:10.1111/jcmm.70083)
Supplement: Supplementary file 4 — Table S1. Primer sequences for prognostic characteristic genes. [file JCMM-28-e70083-s002.pdf]

Supplementary Table 1. Primer sequences for prognostic characteristic genes.

Gene		Primers Sequences
TMCO3	F	TCAAACCATGCTACTCTGTCTT
TMCO3	R	AGCTTGTGAGCAACACAGTT
RAP2A	F	TCATGTGGATCCGAAGGTGC
RAP2A	R	CAGAGCAGCTGAAGGCTGTA
ITGAV	F	GTGCAGCCACTACCCATCTC
ITGAV	R	CGTTCAAACCAGCCAACCAA
ZFYVE26	F	TCCCACGCAAGATGTGACAA
ZFYVE26	R	CTGGCCAGTGAGAGGTTCAG
CHST9	F	CTCCATACAGCTGCCCTTGT
CHST9	R	AGCCAGGAGGCAGAAAATGG
HMGN4	F	GGTGACTTTTGGAGGAGGGG
HMGN4	R	CCCCAAACCAATTTGTATTCAGC
KLHL2	F	CTGAGGAATGGACCTGGTGT
KLHL2	R	CCTGCAGTAATGTTCATGCCA
